# GBR membrane of novel poly (butylene succinate-co-glycolate) co-polyester co-polymer for periodontal application

**DOI:** 10.1038/s41598-018-25952-1

**Published:** 2018-05-14

**Authors:** Seyedramin Pajoumshariati, Hadi Shirali, Seyedeh Kimia Yavari, Sogol Naghavi Sheikholeslami, Ghogha Lotfi, Fatemeh Mashhadi Abbas, Alireza Abbaspourrad

**Affiliations:** 1 Department of Food Science, College of Agriculture and Life Sciences, Cornell University, Ithaca, USA; 2 Department of Polymer Engineering and Color Technology, Amirkabir University of Technology, Tehran, Iran; 3 National Cell Bank of Iran, Pasteur Institute of Iran, Tehran, Iran; 4 Dental Research Center, Shahid Beheshti University of Medical Sciences, Tehran, Iran; 5 Department of Periodontology, Dental School, Shahid Beheshti University of Medical Sciences, Tehran, Iran; 6 Department of Pathology of Shahid Beheshti University of Medical Sciences, Tehran, Iran

## Abstract

In periodontics, osteoconductive biodegradable guided bone regeneration (GBR) membranes with acceptable physico-mechanical properties are required to fix alveolar bone defects. The objectives of the present study were to produce and characterize a novel co-polyester—poly (butylene succinate-co-glycolate) (PBSGL), and fabricate a PBSGL membrane by electrospinning. We then aimed to evaluate the *in vitro* effect of the glycolate ratio on the biocompatibility and osteogenic differentiation of mesenchymal stem cells (MSCs), and evaluate *in vivo* bone regeneration using these membranes in rabbit calvarial defects by histology. Increasing the glycolate ratio of electrospun PBSGL membranes resulted in better cell attachment, greater cell metabolic activity, and enhanced osteogenic potential at both transcriptional and translational levels. Histologic and histomorphometric evaluations revealed further that bone defects covered with fibers of higher glycolate ratios showed more bone formation, with no adverse inflammatory response. These results suggest that novel PBSGL electrospun nanofibers show great promise as GBR membranes for bone regeneration.

## Introduction

Defects in the alveolar bone—the underlying bone structure that holds our teeth together and supports them—can deform facial structure, diminish oral function, and put our dental health at risk. The field of periodontics has sought to correct these defects using guided bone regeneration (GBR) membranes that are placed as a barrier between the bone defect and other tissues to facilitate bone formation^1–3^. In order to achieve their intended purpose, these GBR membranes should be easy to use in dental clinics (*i.e*., manageable and not susceptible to complications) and should attain a balance between their physico-mechanical properties (*e.g*., flexibility, stretch-ability, and space maintenance) and their biological properties (*e.g*., biocompatibility, biodegradability, blood clot stabilization, tissue integration, and bioactivity (*e.g*., osteoconductivity))^4–6^. GBR membranes with osteoinductive and osteoconductive properties can enhance periodontal tissue regeneration better than occlusive barrier membranes as they promote the recruitment and differentiation of progenitor cells—located in the remaining periodontal ligament, adjacent alveolar bone, or blood at the surgical site. Bioactive agents (*e.g*., hydroxyapatite (HA)^7^, nano HA^8–10^, and bioactive glass nanoparticles^11–14^) have been incorporated into GBR membranes to improve their osteoconductivity. These promising studies indicate the necessity of using osteoconductive/osteoinductive materials in manufacturing a new generation of periodontal GBR membranes.

Non-resorbable materials (*e.g*., high-density polytetrafluoroethylene (PTFE) and titanium-reinforced PTFE) and resorbable synthetic or natural-based polymers have been used for GBR membranes^3^. While non-resorbable materials have excellent biocompatibility, they require a second surgery^3,15^, and cause the risk of losing some of the regenerated bone^16^. In contrast, resorbable materials are more favored because they reduce the risk of loss of newly formed bone and do not require a second surgical procedure^3,15,17^. There is a tradeoff between biological properties and physical characteristics of today’s biodegradable materials. The two major types of biodegradable materials currently being used for these membranes (collagenous materials and synthetic polymers) do not quite achieve this balance. Collagenous membranes, such as Bio-Gide, for example, show good biological properties but poor physico-mechanical characteristics. They are highly biocompatible^8^ (*i.e*., they show good bio-affinity and resorbability^18^, and decreased tissue morbidity^19^), but have poor mechanical properties and a high degradation rate—*i.e*., they do not shield the bone defect efficiently^20^ and collapse before the bone has time to heal^21^, thus not allowing enough space for the blood clot to transform into bone^17^. Before they can be fully functional, collagenous membranes need to be further crosslinked, delaminated, or blended with other polymers. Consequently, there is considerable heterogeneity in the physical qualities of collagenous membranes used in clinical practice based on their construction. These variations (*e.g*., collagen crosslinking process) can alter both handling characteristics and degradation time of collagenous membranes.

Synthetic polymers generally exhibit the opposite issue: they have good mechanical properties, but poor biological characteristics^3^. Biodegradable, aliphatic polyesters, such as poly (glycolic acid) (PGA), poly (lactic acid) (PLA), poly (lactide-co-glycolide), poly (lactide-co-caprolactone), and poly (butylene succinate) (PBS), have been extensively studied for this purpose. PGA, the simplest member of this family, with a moderate degree of crystallinity, has good biocompatibility, but it cannot support tissue regeneration because it degrades quickly inside the mouth by hydrolysis, specifically when it is in the form of electrospun nanofibers. In addition, *in vivo* applications of PGA have resulted in acidosis^22^, fibrous capsule formation, and foreign body reaction^23^. PBS is an alternative polyester that has been used due to its favorable mechanical properties and excellent processability. Compared to PGA, PBS’s lower hydrolytic degradation rate and harmless degradation products (CO_2_ and H_2_O) would make it a better candidate for bone tissue engineering, were it not for its poor biological properties (*i.e*., poor *in vitro* biocompatibility and *in vivo* osteo-compatibility)^24^. Attempts have been made to address this limitation. For example, Chen *et al*. recently synthesized poly(butylene succinate/diglycolate) by melt-mixing PBS with poly(butylene diglycolate) and showed that this co-polyester had an osteogenic potential superior to that of the polyester with thio-ether (butylene thiodiglycolate)^25^. The current challenge is to make new, biodegradable materials with tunable mechanical properties that are also biocompatible and osteoconductive. These new materials would serve as an effective barrier membrane between the surrounding soft tissue and the bone defect during bone regeneration, and then—when the bone is healed—degrade on its own at an appropriate rate.

With this in mind, in the present study we synthesized novel co-polyesters based on PBS and PGA by esterification of a diol (bis(4-hydroxybutyl) succinate (BHBS)) and di-acid (polyglycolic acid (PGL)) with varying glycolate ratios. This co-polymerization would bring together the biocompatibility of PGA and the good mechanical properties of PBS to make a co-polyester with adjustable hydrolytic degradation. The thermal and mechanical properties of these novel co-polyesters, as well as their microstructures, were investigated. These co-polyesters were then electrospun to fabricate non-oriented, nanofibrous membranes with high surface area, better mechanical strength, and biomimetic properties.

The biological activity, as well as the osteoconductivity, of four fabricated GBR membranes with different glycolate ratios was evaluated *in vitro*. In addition, their osteoconductivity and new bone formation potential were analyzed using a rabbit calvarial defect model, a standard model for the GBR technique^26^. This model, which provides enough size for surgical access and handling, as well as support for implanted materials without any need for fixation due to its proper anatomic location, has been accepted as a good predictor for bone regeneration in small to moderate bone defects^26^.

## Results and Discussion

### Synthesis and characterization of co-polyesters

**Figure 1:**
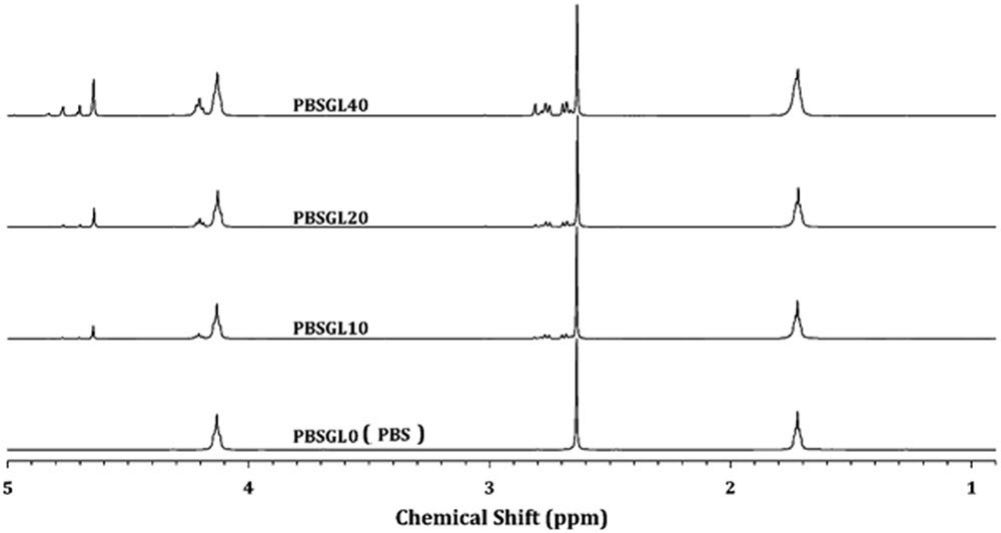
^1^HNMR spectra of PBSGL_n_ samples.

PBSGL_n_ co-polyesters with different mole percentages of PGL (n) were produced according to the experimental section and summarized in Table 1. ^1^HNMR spectra of the nanocomposites are shown in Fig. 1 and were used to investigate their structure. We assigned the shifts as: ^B^H_b_ (1.72 ppm), ^S^H_a_ (2.64 ppm), ^B^H_a_ (4.13–4.22 ppm) and ^G^H_a_ (4.63–4.76 ppm)^27,28^, where ^S^H, ^B^H and ^G^H represent the hydrogen atoms of succinic acid (SA), butylene glycol (BG) and glycolide (GL), respectively, and the subscripts are shown in Fig. 1. The GL mole fraction in the polymer chain was calculated according to the following equation:

1 $$GL\,mole\,fraction=\frac{{G}_{{H}_{a}}}{{G}_{{H}_{a}}+{S}_{{H}_{a}}}$$

**Table 1:** Characteristics and ^1^HNMR results of the synthesized co-polyesters.

Sample	GL mole fraction in feed	GL mole fraction in co-polyester	L_nBGl_	L_nBSu_	R	[η] (dL/g)	M_n_ (g/mol)	COOH (meq/kg)
PBSGL0	0	0	—	—	—	1.41	55846	25
PBSGL10	10	12.3	1.8	22.8	0.59	1.24	45821	18
PBSGL20	20	21.6	1.9	16.5	0.65	1.29	48697	15
PBSGL40	40	39.4	2.1	9.0	0.58	1.24	45821	20

The number-average sequence lengths of butylene succinate (BSu) and butylene glycolate (BGl) units (L_nBSu_ and L_nBGl_, respectively) as well as the randomness (R) were calculated following the procedure described in our previous publication^29^. The co-polyester is produced when the value of R is close to 1.0. The results are shown in Table 1. The BGl mole fraction in the polymer chain and feed were slightly different, and increasing the BGl mole fraction led to a decrease in BSu sequence length. The randomness of the co-polyesters was almost the same, which indicates that changing the mole fraction does not affect it.

**Figure 2:**
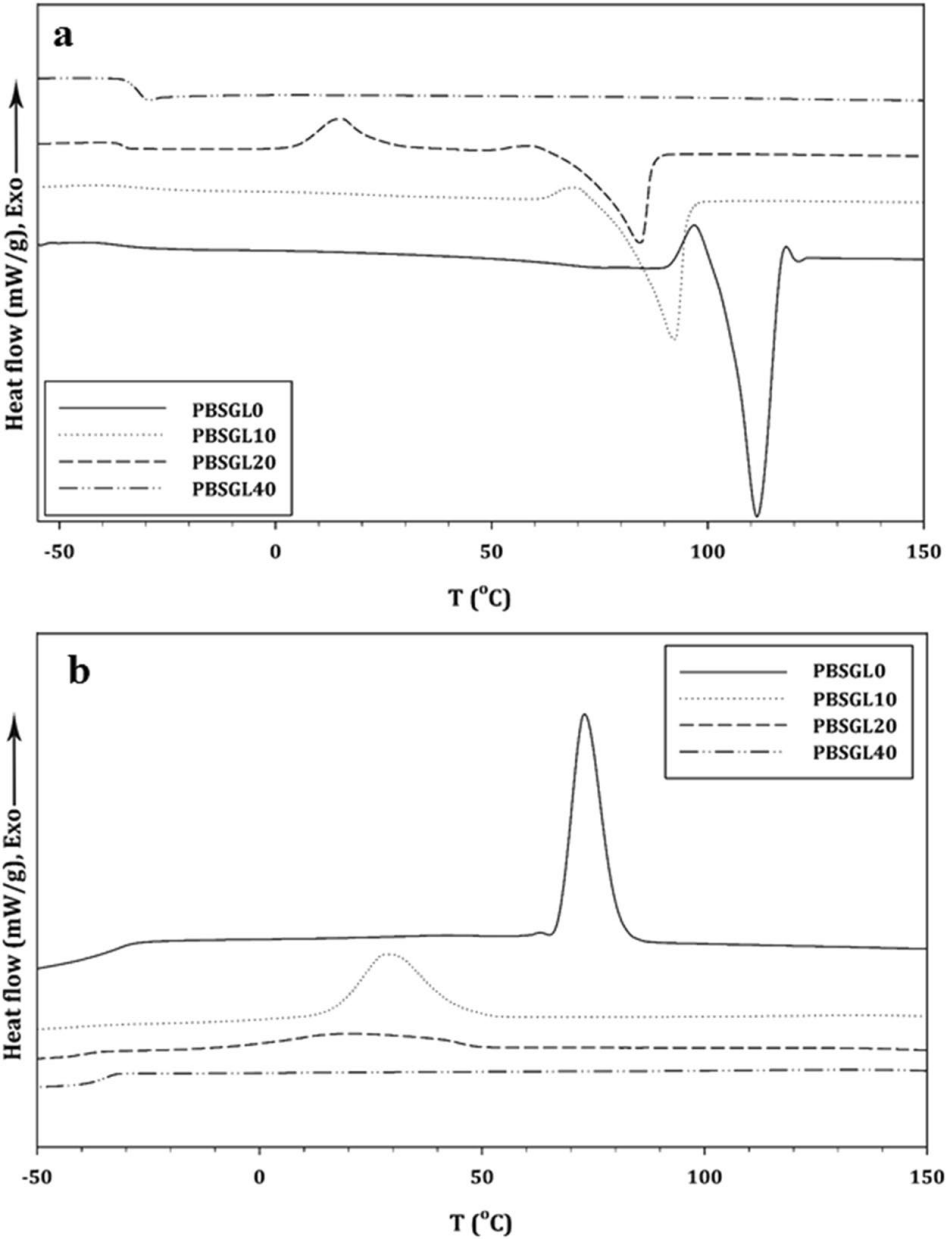
(**a**) Heating and (**b**) cooling DSC thermograms of PBSGL_n_ polyesters at rates of 10 °C/min, each.

Heating and cooling differential scanning calorimetry (DSC) thermograms of the co-polyesters are shown in Fig. 2 and related results are summarized in Table 2. The synthesized co-polyesters showed a single glass transition, which slightly increased with the GL mole fraction. This means that the BGl segment was harder than BSu, which restricts chain motion and leads to an increase in T_g_^30^. A melting point (T_m_) existed in the heating thermogram of the co-polyesters, decreased and broadened with increasing the ratio of the co-monomer to the polymer, and finally was removed by introducing 40 mole% BGl. These results indicate that the crystallization behavior can be changed by introducing a structurally-different second co-monomer which hinders crystallization^29^. The structure and concentration of the second co-monomer are two important parameters, which can allow the second co-monomer to participate in crystal lattice formation^31^. Baur’s equation was fitted to clarify the presence of BGl in the crystal lattice^32^:

2 $$\frac{1}{{T}_{m}}=-\,\frac{R}{{\rm{\Delta }}{H}_{u}}\,\mathrm{ln}({x}_{a})+\frac{1}{{T}_{m}^{0}}=-\,0.0125\times \,\mathrm{ln}({x}_{a})+0.01,\,{R}^{2}=0.984$$

in which *ΔH*_*u*_*, R, T* ^0^_*m*_ and *x*_*a*_ are the gas constant, enthalpy of fusion, equilibrium melting temperature, and main monomer composition, respectively. The results showed that BGl did not participate in lattice formation. Moreover, ^1^HNMR results showed that the BSu sequence length decreased with co-monomer content. Therefore, crystallite size decreased with the introduction of a second co-monomer. Equation 3 was used to calculate the degree of crystallinity (χ_c_) for all samples:

3 $${\chi }_{c}=(\frac{{\rm{\Delta }}{H}_{m}}{{\rm{\Delta }}{H}_{mPBS}^{0}\times {\varphi }_{1}+{\rm{\Delta }}{H}_{mPGL}^{0}\times {\varphi }_{2}})\times 100$$

in which ΔH^o^_mPBS_ and ΔH^o^_mPGL_ are the heats of fusion for 100% crystalline poly(butylene succinate) and polyglycolide, which are 210 and 139 J/g, respectively^33,34^, *Ø*_1_ and *Ø*_2_ are the weight fractions of BSu and BGl, respectively, and ΔH_m_ is the heat of crystallization of the co-polyesters. The results (Table 2) indicated that the degree of crystallinity decreased with increasing BGl.

**Table 2:** DSC and XRD results of the PBSGL_n_ co-polyesters.

Sample	T_g_ (°C)	T_c_ (°C)	ΔT_c_ (°C)	$\frac{\Delta Hc}{Time}(J/gs)$	ΔH_m_ (J/g)	T_m_ (°C)	ΔT (°C)	*χ*_*c* (%)_	N	Z_t_	L_002_ (nm)	L_110_ (nm)	L_110_ (nm)
PBSGL0	−34.5	73.2	21.9	0.57	75.8	111.9	38.7	36.1	3.43	0.61	15.2	12.1	—
PBSGL10	−33.8	28.5	39.9	0.20	51.1	92.1	63.6	33.0	3.67	0.154	11.2	6.5	13.5
PBSGL20	−32.2	19.2	56.4	0.07	34.9	84.3	65.1	25.4	3.80	0.11	9.3	4.2	12.9
PBSGL40	−31.5	—	—	—	—	—	—	—	—	—	2.7	—	6.1

A crystallization peak could be seen in the cooling DSC thermogram, which followed the same trend as T_m_ with the incorporation of the co-monomer. Lower degrees of super-cooling (ΔT = T_m_ − T_c_), a smaller crystallization peak width, and a higher ΔH_c_ per time of crystallization indicated a higher overall crystallization rate^35^. These parameters are represented in Table 2 and confirm that the overall crystallization rate decreased with co-monomer content. Moreover, the PBSGL20 thermogram showed a cold crystallization peak at about 15 °C, which indicates that the crystallization rate was lower than cooling.

**Figure 3:**
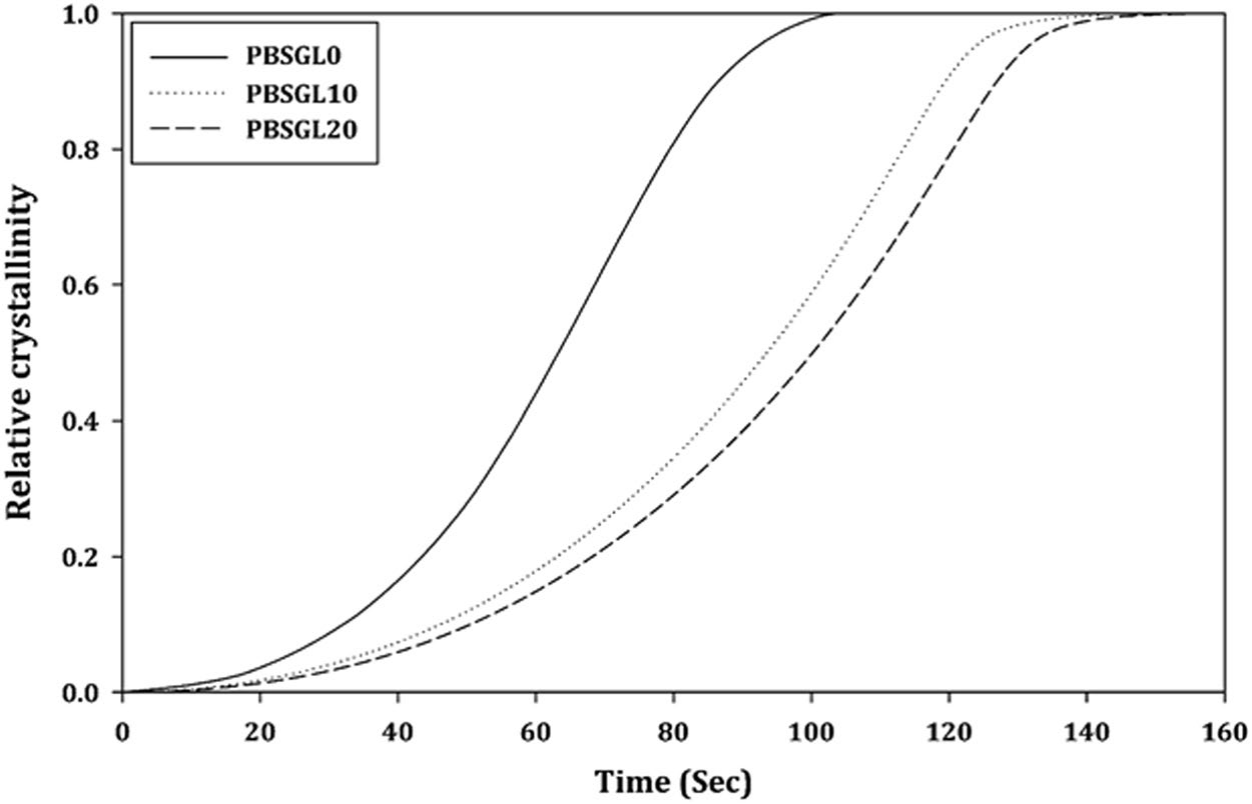
Development of non-isothermal relative crystallinity over time for PBSGL_n_ co-polymers.

Non-isothermal crystallization was studied by non-linear curve fitting of the Ozawa equation to the relative crystallinity (*X*_*t*_) data calculated from the DSC results (Fig. 3)^36^:

4 $${X}_{t}=1-\exp ({Z}_{t}{t}^{n})\,\& \,t=\frac{{T}_{o}-T}{C}$$

in which *T*_0_*, n, Z*_*t*_, and *C* are the onset temperature of crystallization, Ozawa power, growth rate constant, and cooling rate, respectively. The results (Table 2) indicate that the crystals of all co-polymers had spherical forms. Moreover, Z_t_ values decreased with co-monomer content, confirming the decrease of the crystallization rate.

**Figure 4:**
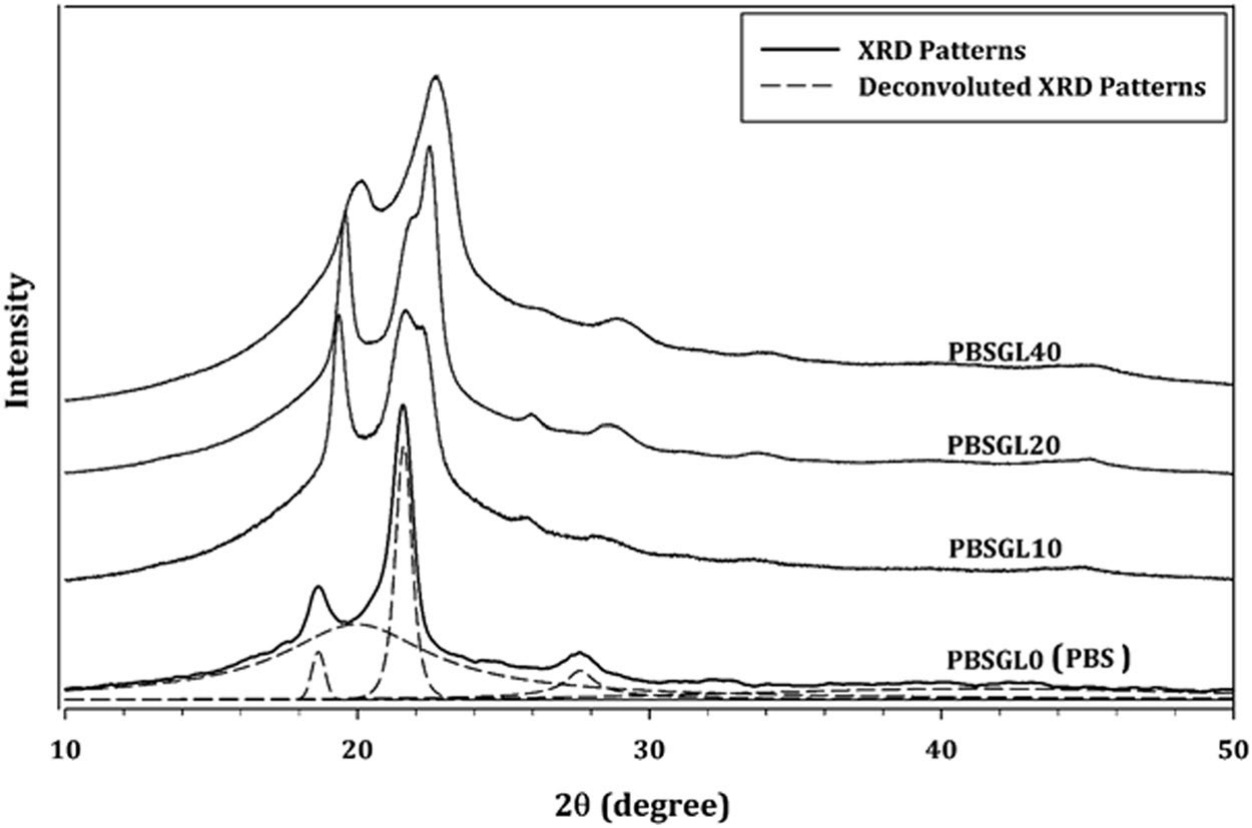
XRD patterns of PBSGL_n_ co-polyesters.

X-ray diffraction (XRD) patterns of all co-polyesters are presented in Fig. 4. Three peaks can be seen in the PBSGL0 XRD pattern at 2θ ≈ 18.7, 21.6, and 27.7°, which were attributed to (002), (110), and (111) planes, respectively^37^. The peaks shifted to higher angles and a new peak at 2θ ≈ 22.4° appeared by increasing the BGl content in the co-polymers, which indicates that the crystallite size decreased and a new crystal plane was formed, which was attributed to the (110) plane of PGL^38^. The Pearson VII equation was used to deconvolute the diffraction peaks (dashed lines). The results show that the width at half of the maximum peaks (B) increased with co-monomer content. The Scherrer equation was used to calculate mean crystal sizes of the “hkl” plane (L_hkl_)^39^:

5 $${L}_{hkl}=\frac{K\lambda }{B\,\cos (\theta )}$$

in which K and λ are the Scherrer factor (0.9) and peak position, respectively. The results (Table 2) confirmed that the crystallite sizes decreased with increasing BGl content, indicating that the structurally different co-monomer hindered crystallization.

### Electrospinning of PBSGL co-polyesters

**Figure 5:**
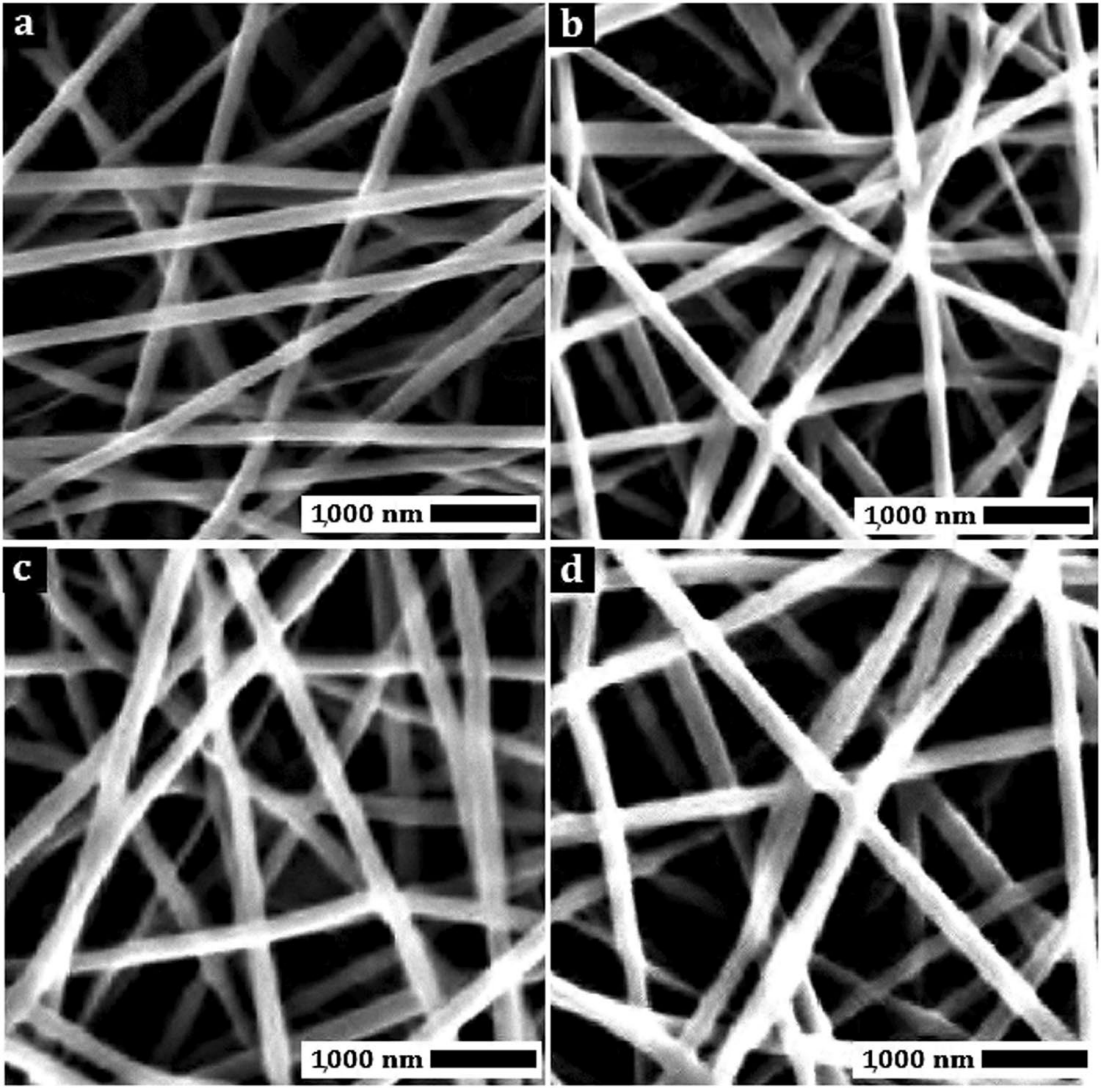
SEM images of (**a**) PBSGL0, (**b**) PBSGL10, (**c**) PBSGL20, and (**d**) PBSGL40 nanofibers.

Electrospinning parameters were varied to fabricate co-polyester nanofibers featuring the lowest mean fiber dimeter (FD) and standard deviation (STD). Nanofiber scanning electron microscopy (SEM) images were used to calculate FD and STD. The results showed that an electrospinning solution concentration of 11 wt%, an applied voltage of 20 kV, and needle-to-collector distance of 9 cm were the optimal parameters to achieve a low mean FD and STD of the resulting nanofibers. Figure 5 shows SEM images of co-polyester nanofibers, produced at optimal conditions. The FD and STD of these co-polyester fibers are presented in Table 3. As can be seen, bead-free and continuous nanofibers were produced using these conditions.

**Table 3:** Nanofiber fabrication characteristics, tensile strength, wettability, and *in vitro* hydrolysis degradation results of the PBSGL_n_ co-polyesters.

Sample	FD (nm)	STD (nm)	E (MPa)	σ (MPa)	Elongation @ break (%)	Contact angle (°)	*In-vitro* mass loss after 60 days
PBSGL0	215	68	74.7	4.6	13.1	97.8 ± 4	3%
PBSGL10	210	57	42.4	4.0	17.2	88.2 ± 5	7%
PBSGL20	208	46	22.9	2.5	44.2	77.4 ± 3	9%
PBSGL40	205	61	14.8	1.4	12.8	63.8 ± 5	13%

**Figure 6:**
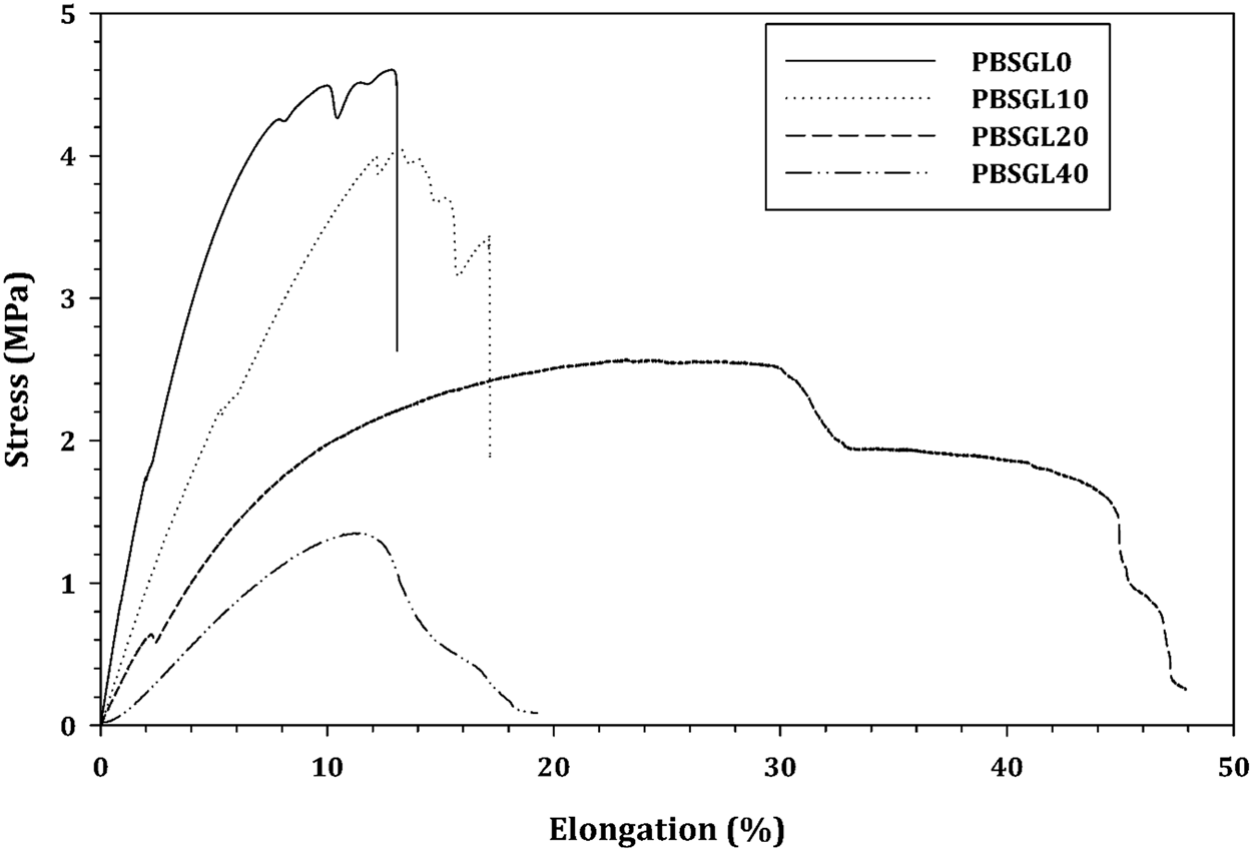
Tensile stress-strain curves of PBSGL_n_ electrospun nanofibers.

Tensile stress-strain results of the PBSGL_n_ electrospun nanofiber membranes and the quantitative data derived from these graphs are shown in Fig. 6 and Table 3, respectively. The tensile strength and the elastic modulus decreased with the BGl content, but elongation at break first increased then decreased. An increase in crystallization degree, or mixing the polymer with hard particles, leads to an increase in tensile strength and elastic modulus^40^. As mentioned before, the incorporation of BGl content into co-polyesters led to decreased crystallinity. Therefore, increasing the soft BGl content and decreasing crystallinity reduced both tensile strength and elastic modulus. Moreover, this increase resulted in an increase in surface wettability/hydrophilic properties of the PBSGL_n_ membranes analyzed by water contact angle measurements. Table 3 shows that increasing the PGL ratio up to 40% (PBSGL40) resulted in a 53% decrease in water contact angle and consequently an increase in wettability and hydrophilicity of the pristine PBS (PBSGL0). The degradation rate (mass loss) after 60 days (Table 3) indicates that an increase in the PGL ratio of the PBSGL membranes resulted in an increase in degradation rate in a concentration-dependent manner.

### MSC morphology and proliferation/metabolic activity on fabricated membranes

**Figure 7:**
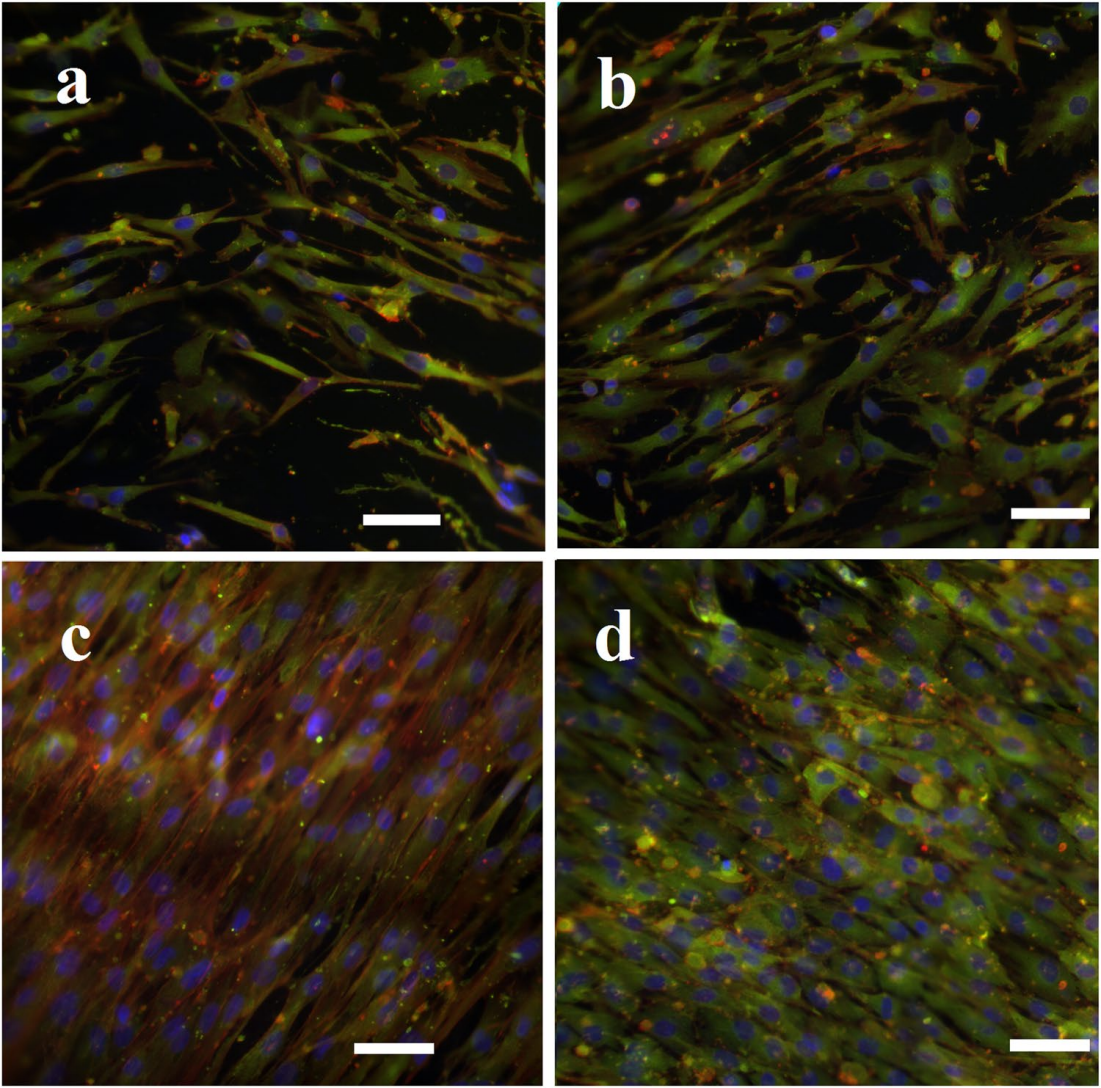
Morphology of rabbit MSCs on PBSGL nanofibers (PBSGL0 (**a**), PBSGL10 (**b**), BSGL20 (**c**) and PBSGL40 (**d**)) seeded at a cell density of 4000 cells per cm^2^ after 7 days’ culture. Cells were immuno-stained with an antibody against vinculin (green), FITC–phalloidin (red), and DAPI (blue). Scale bar = 100 μm.

To evaluate the morphology of MSCs on PBSGL_n_ fibers, cytoskeletal actin filaments, as well as vinculin as a key focal adhesion protein, were stained (Fig. 7). Increasing the PGL ratio resulted in facilitated adhesion of MSCs with well-spread and elongated morphologies, specifically for PBSGL20 and PBSGL40 nanofibers, which can be attributed to the higher hydrophilic characteristics of the materials^22^. It is established that hydrophilic surfaces can enhance cellular adhesion^41^. For all PBSGL_n_ fibers, green fluorescent-stained vinculin proteins were intensely visible in the MSCs cultured on them. Comparing the intensity of green fluorescent stains between PBSGL20 and PBSGL40 indicated a higher vinculin-anchored focal adhesion of the latter group (Fig. 7). Moreover, cytoskeleton reorganization was more apparent in PBSGL_n_ fibers with a higher ratio of PGL.

The improved hydrophilic properties and better cell adhesion observed in the current study, as compared to other polyesters based on butylene succinate and diglycolate (*e.g*., Chen *et al*.^25^), can be attributed to greater ester bond formation in the repeating unit of the PBSGL_n_ polyester. These ester bonds can further be subjected to hydrolysis *via* exposure to carboxylic acid and hydroxyl groups. Two important parameters that affect cellular adhesion are surface chemistry and topography. Because PBSGL_n_ fibers possess similar topographic features, the difference in adhesion properties would be mainly due to differences in surface chemistry and functional groups. It has been reported that the presence of C–OH groups on the PBS polyester could result in higher osteoblast adhesion and proliferation^42^. In this study, we showed that increasing the PGL ratio, and consequently the hydrophilicity, of PBSGL_n_ fibers results in higher cellular adhesion and proliferation.

The metabolic activity of rabbit MSCs on PBSGL0, PBSGL10, PBSGL20 and PBSGL40 membranes increased 5.7-, 5.9-, 7.2-, and 7.6-fold, respectively, from day 1 to day 12. A two-way analysis of variance (ANOVA) showed that both PGL content and day did have a significant effect on the cellular metabolic activity (*i.e*., the cell proliferation rate) (F (3,32) = 3.87, P < 0.047 and F (3,32) = 7.26, P < 0.008). The interaction of PGL content and day was also significant (F (9,32) = 2.15, P < 0.05). Pairwise comparison between days showed that there were significant differences for cell viabilities between day 1 and day 7 as well as day 1 and day 12 (P < 0.05 and P < 0.03, respectively). However, there was no significant difference between day 7 and 12.

**Figure 8:**
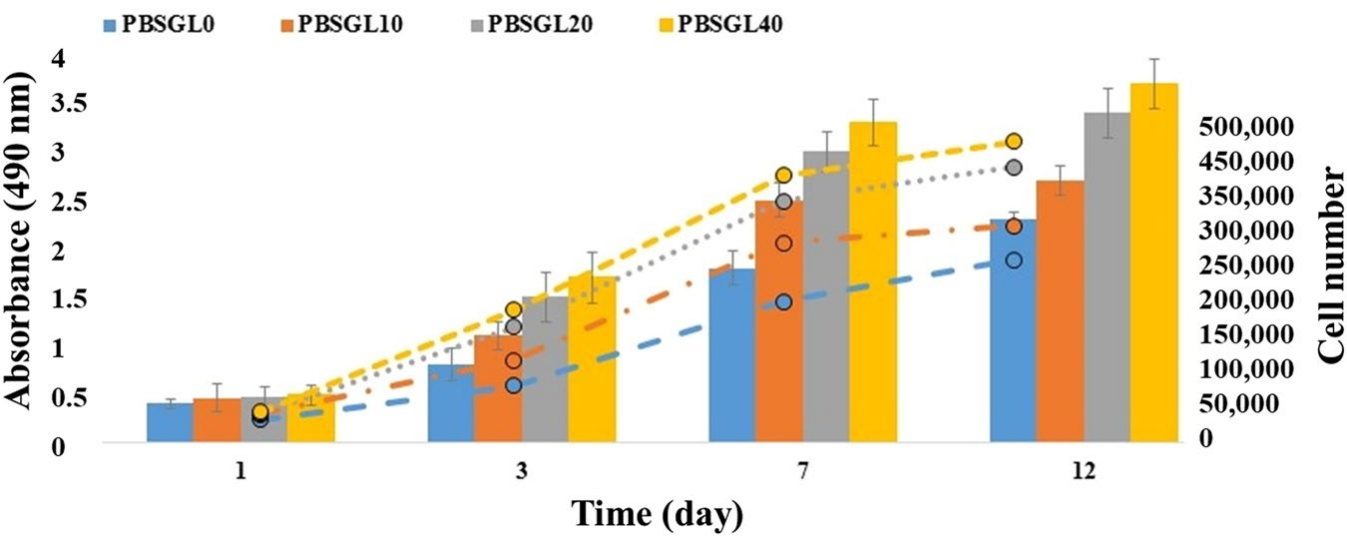
Metabolic activity (proliferation) of rabbit MSCs cultured on electrospun nanofibers submerged in basal medium at day 1, 3, 7, and 12. The secondary y axis shows the number of rabbit MSCs for colored dashed lines corresponding to each group. An asterisk denotes a statistically significant difference (P < 0.05) between the test and control group at each time point. The sample size selected was 5.

The results of the rabbit MSC metabolic activity (proliferation, shown in Fig. 8) revealed that the cells were metabolically active on all PBSGL_n_ membranes. During the first week of culturing rabbit MSCs on PBSGL_n_ membranes, the number of cells increased at a higher rate compared to day 12, as these cells had more surface area on the membranes available for growth (Fig. 8).

The metabolic activity (proliferation) of MSCs on PBSGL_n_ membranes with higher ratios of PGL was found to be considerably higher than those with lower ratios of PGL as well as for the pristine PBS (PBSGL0) membrane, which stems from the higher hydrophilicity and wettability. This increase in proliferation can be partially attributed to a higher degree of vinculin-anchoring focal adhesion, which potentially enhances the cellular signaling involved in MSC proliferation^43^.

### Osteogenic differentiation

**Figure 9:**
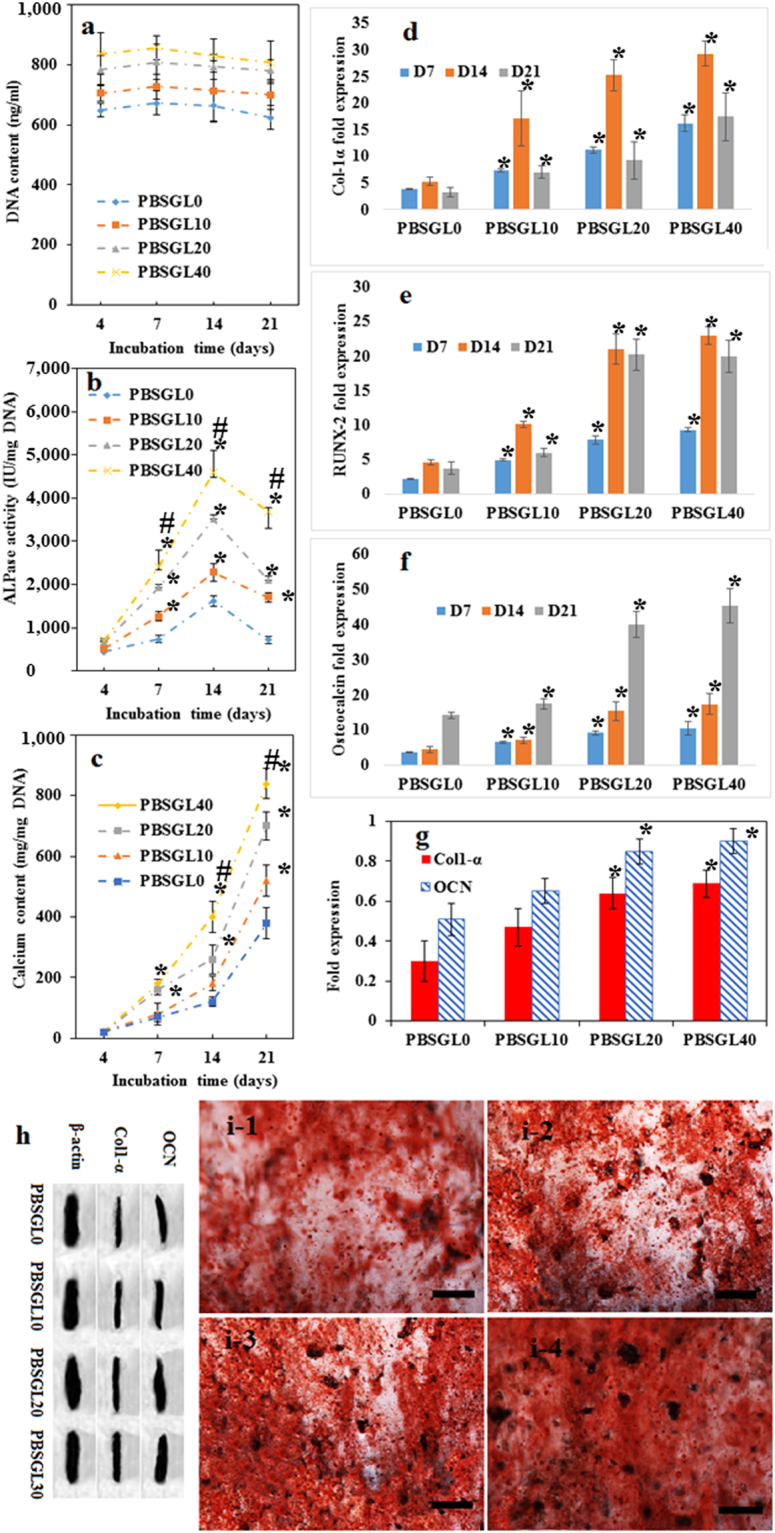
Osteogenic differentiation of rabbit MSCs cultured on electrospun PBSGL_n_ membranes at days 4, 7, 14, and 21 in the osteogenic media: DNA (**a**); ALP activity (**b**); calcium content (**c**); gene expression (Col-α1 (**d**), Runx-2 (**e**), and OCN (**f**)); protein expressions of OCN and Col-α1 at day 21 relative to β actin expression (**g**); Western blotting bands for OCN (12 kDa), Col-α1 (130 kDa), and β actin (42 kDa) (h). Three different gels (with the same acquisition settings and exposure parameters) were used to visualize the relative protein expressions of OCN, Col-α1 and β actin proteins. Alizarin Red staining showing deposited calcium ions on the electrospun PBSGL0 (i-1), PBSGL10, (i-2) PBSGL20, (i-3) and PBSGL40 (i-4) nanofibers at day 21. An asterisk denotes a statistically significant difference P < 0.05) between the test and control group at each time point. A “#” sign indicates a statistically significant difference between the test and all other groups at each time point. Sample size selected was 5.

To normalize the values for alkaline phosphatase (ALP) activities and calcium content, we measured DNA content of cell-seeded membranes using the PicoGreen DNA assay. The results showed that there was no significant change in DNA content of rabbit MSCs seeded on PBSGL_n_ membranes cultured in osteogenic medium with incubation time (Fig. 9a). Furthermore, ALP activities of MSCs seeded on the PBSGL_n_ membranes followed its common trend in osteogenesis^44^ (an increase from day 7 to 14 related to osteogenic differentiation followed by a decrease from day 14 to 21 related to the initiation of the mineralization process, Fig. 9b). As can be seen, the normalized amount of ALP activity, which has a major role in mineralization and the formation of hydroxyapatite crystals, increased with the increasing ratio of PGL in PBSGL_n_ membranes. An increase in the ALP activity of PBS substrates^45^ and plasma-treated PBS films^46^ (for the first two weeks) have been previously reported. It was shown that plasma-treated PBS films with higher hydrophilicity improved ALP activity as well as the cell viability of rat calvaria osteoblasts^46^. The calcium content of seeded rabbit MSCs, on the other hand, increased with incubation time (Fig. 9c) as well as with increasing PGL ratio. The PBSGL40 membrane possessed a significantly higher calcium content compared to other groups at day 14 and 21 (P < 0.05).

Osteogenic gene expressions (Col-α1, Runx-2, and Osteocalcin (OCN)) of rabbit MSCs cultured on PBSGL_n_ membranes (Fig. 9d–f) indicate that membranes with higher ratios of PGL could upregulate these genes. A two-way analysis of variance revealed that the PGL ratio had a significant effect on the differentiation of the MSCs (F (3,32) = 3.41, P < 0.032). Statistical analysis showed a significant difference between the groups with higher amounts of PGL content (PBSGL40 and PBSGL20) and other groups (P < 0.02) for OCN, Col-α1, and Runx-2 gene expression at each time point; however, no statistically significant difference was found between the PBSGL40 and PBSGL20 groups. The statistically significant, higher osteogenic potential of PBSGL_n_ fibers with higher ratios of PGL (PBSGL20 and PBSGL40)—which induced osteogenic differentiation at the translational level in rabbit MSCs—was confirmed with Western blot analysis of OCN and Col-α1 proteins at day 21(Fig. 9g and h). Consequently, it can be said that the higher amount of PGL in PBSGL40 and PBSGL20 groups can transcriptionally and translationally improve the osteogenic differentiation potential of PBSGL fibers.

Alizarin Images of red-stained rabbit MSCs seeded on PBSGL_n_ nanofibers at day 21 confirmed the calcium content results (Fig. 9i1–4). The PBSGL20 and PBSGL40 groups showed a relatively high density of orange-red areas, qualitatively confirming that these groups contained a higher calcium content compared to the PBSGL0 and PBSGL10 groups (Fig. 9i1–4). Bone nodule structures were formed on all groups with PGL—shown as large dark spots (>35 µm^47^) in the Alizarin Red staining images with a number density of 187, 142, 121 counts/cm^2^ and a total area of 5.8 ± 0.5, 4.6 ± 0.3 and 3.4 ± 0.4 mm^2^/cm^2^ for the PBSGL40, PBSGL20, and PBSGL10 samples, respectively.

The enhanced, concentration-dependent osteogenic differentiation of seeded MSCs can be attributed to the improved surface wettability and hydrophilicity of PBSGL_n_ fibers with increasing PGL ratio^48^. Suphakit *et al*. showed that by hydrolyzing PBS surfaces in order to increase their hydrophilicity and wettability, the bioactivity and osteogenic differentiation potential of PBS polyesters can be improved significantly^49^.

### *In vivo* evaluation of bilayer membranes

Using histological methods, the amount of new bone formation was shown to be related to the PGL ratio of the PBSGL_n_ membranes that covered the defect. The amount of new bone formations (Table 4) revealed that more bone was formed in PBSGL40 and PBSGL20 groups than in PBSGL10 and PBSGL0. The defects covered with PBSGL40 showed the highest amount of bone formation in all directions, while the coverage for other groups was not complete. There was a statistically significant main effect of bone formation for surgical groups (F (3, 24) = 12.514, P < 0.001). The extent of new bone formation for the PBSGL0 control group was significantly different from that of PBSGL40 (F (1, 8) = 21.147, P < 0.01) and PBSGL20 (F (1, 8) = 17.103, P < 0.04) as well as PBSGL10 (F (1, 8) = 13.392, P < 0.05). The amount of new bone formation for PBSGL10 was significantly different from that of PBSGL20 (F (1, 8) = 4.132, P < 0.05) and PBSGL40 (F (1, 8) = 7.625, P < 0.02). The amount of bone formation for PBSGL20 and PBSGL40 showed no significant difference (F (1, 8) = 2.850, P = 0.06). There was no significant main effect for time (F (1, 8) = 4.088, P = 0.08), though there was an interaction effect for time and the surgical groups (F (3, 24) = 0.478, P = 0.531). Based on our previous report, the amount of new bone formation by percentage for similar calvarial defects on rabbits that received no covers (negative control) after one and two months were 15% and 21%, respectively^4^. Results of the present work revealed that the presence of PBSGL_n_ membranes improved bone formation in a PGL concentration-dependent manner (22%, 34%, 75%, and 85% at day 30 and 29%, 44%, 91%, and 96% at day 60 for PBSGL0, PBSGL10, PBSGL20, and PBSGL40, respectively). The results obtained for PBSGL20 and PBSGL40 membranes were also comparable with the bone formation results obtained for similar calvarial defects covered by a Bio-Gide membrane as a positive control, with 96% coverage after one month^4^.

**Table 4:** The new bone formation in the surgical groups at two time points after surgery (mean ± standard error).

	Newly-formed Bone (mm^2^)		
	Time	One Month After Surgery	Two Months After Surgery
Surgical Groups	PBSGL0	6.24 ± 5.27	8.32 ± 5.65
	PBSGL10	9.74 ± 4.92^*^	12.33 ± 5.97^*^
	PBSGL20	21.14 ± 4.34^*^	25.78 ± 3.14^*^
	PBSGL40	24.15 ± 2.18^*^	27.13 ± 1.08^*^

(One star denotes a statistically significant difference (P < 0.05) between the control group and the test at each time point. n = 5 animals at each time point. Each animal had 4 calvarial defects and from each defect 6 histological cross-sections were used).

The Friedman test revealed that there was no statistically significant difference between the inflammatory response of PBSGL_n_-surgically-covered defects (χ2 (3) = 6.132, P = 0.158) at two time points (one and two months after surgery), and the assigned inflammation grade reported by a histologist was zero (less than 10 inflammatory cells per field in each group). This mild immune response can potentially be related to the small size of the PBSGL_n_ fibers^22,50^, as well as the slow degradation rate of the PBSGL_n_ membranes, and consequently the slight change of pH in the surgical areas, in contrast to PGA fibers, which feature a fast degradation rate that can elicit an immune response^51^. It is worth mentioning that polymer type and its chemistry can also play a major role in immune response. For instance, PBS alone and its blend with PLA have shown an alleviated immune response compared to the PLA after a month^24^.

There are a few studies examining the *in vivo* application of PBS-based polymers for bone healing. This includes studies involving the femoral bone of rabbits for a period of one and two months^52^, mice calvaria (two months)^53^, and iliac submuscular regions and cranial defects of Wistar rats (three months)^54,55^. Niu *et al*. observed that incorporation of nano-fluorapatite (nFA) into PBS resulted in improved hydrophilicity of pristine PBS polyester, as well as improved cellular behavior and osteogenic differentiation of human MSCs^52^. The osteoconductivity of nFA/PBS nanocomposites was confirmed by evaluation of new bone formation in the femoral bone of rabbits, as well as the absence of fibrous capsules in the surrounding tissue for a period of one and two months^52^. Chitosan has also been used to improve bioactivity and osteoinductivity of PBS scaffolds^53–55^. Critical-size, cranial bone defects that were covered by chitosan-containing scaffolds exhibited higher osteo-integration and a milder inflammatory response for a period of two months after surgery compared to the pure PBS scaffold^53,54^. In a similar study, this group also showed that the normal inflammatory response to the chitosan-containing PBS scaffolds was accompanied by increased vascularization and collagen deposition over time^55^.

Our results showed that these novel PBSGL_n_ co-polyesters with tunable physicomechanical properties were produced by a polycondensation reaction between a diol (BHBS) and di-acid (PGL). The increase in the PGL ratio can improve the hydrophilicity of the PBSGL_n_ polyesters and consequently the cellular behavior of MSCs cultured on them in terms of adhesion, proliferation, and osteogenic differentiation. The increase in metabolic activity (proliferation) with increasing glycolate content can partially be due to the increased surface hydrophilicity of the electrospun fibers (up to 53% decrease in contact angle for PBSGL40), which can promote cell adhesion and proliferation by facilitating nutrient transportation to the seeded cells^41^. Cell spreading, proliferation, and differentiation have also been related to surface hydrophilicity in addition to initial attachment of the cells^25,42,43,46,52,56^. For example, rat calvaria osteoblasts on the hydrophilic surface of plasma-treated PBS films showed accelerated metabolic activity and improved osteogenic differentiation compared to the untreated one^46^. Here we showed that by increasing the ratio of PGL in PBSGL_n_ nanofibers, the hydrophilicity of the membranes significantly increased.

Gene expression of collagen type 1-α (col-1-α; involved in extracellular matrix (ECM) synthesis and bone mineralization^57^), runt-related transcription factor (RUNX-2; involved in osteoblastic differentiation and bone formation^58–60^), and OCN (involved in terminal osteoblastic differentiation and bone mineralization^61,62^), as well as protein expression for col-1-α and OCN, was evaluated to assess the osteogenic differentiation potential of PBSGL_n_ membranes and the effect of the glycolate ratios. The expressions of the RUNX-2 gene increased sharply for all groups from day 7 to 14, which can be related to early stage osteogenic differentiation of rabbit MSCs^58^. The sharp increase in OCN expression as the most abundant non-collagenous protein in bone ECM, between day 14 and 21, can be related to the observed RUNX-2 gene profile, since RUNX-2 is able to directly stimulate the transcription of the OCN gene^60^. It is worth mentioning that up-regulation of OCN is essential for terminal osteogenic differentiation and the formation of minerals^61^. The presence of bone nodules—where MSCs at three stages, including proliferation, differentiation, and mineralization, are simultaneously presented^47^—indicated the final stage of osteoblastic differentiation of MSCs on PBSGL_n_ fibers. Moreover, protein expression of OCN and collagen (Fig. 9g) can explain the improved mineralization on the PBSGL_n_ membranes observed in Alizarin Red staining.

**Figure 10:**
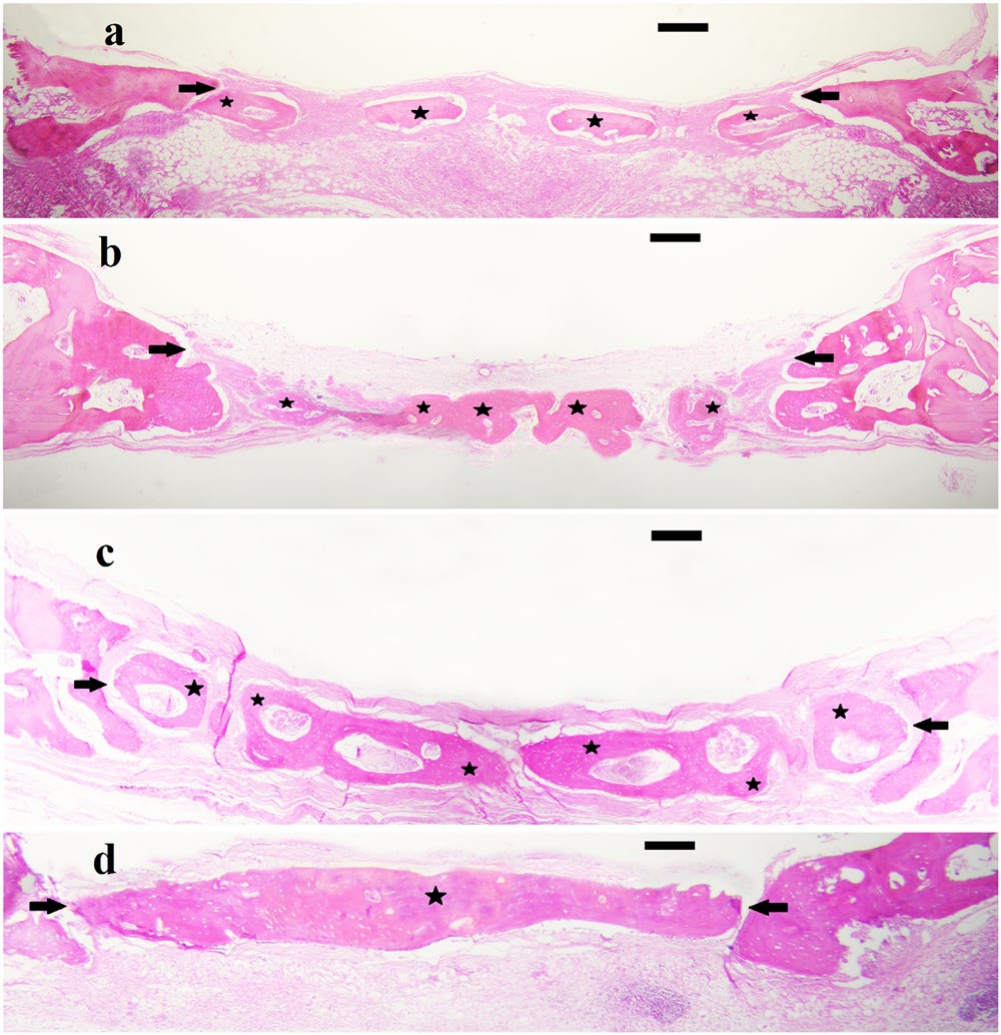
Hematoxylin and eosin staining of membrane coated defects using (**a**) PBSGL0 control, (**b**) PBSGL10, (**c**) PBSGL20, and (**d**) PBSGL40. The stars indicate the amount of newly formed bone two months after surgery in the surgical margins. Original magnification is ×40. Scale bars = 500 µm.

The observed gene expression profile for rabbit MSCs seeded on the PBSGL0 control group in osteogenic medium was in line with other studies^45,46,49^. Our results showed that the novel PBSGL_n_ co-polyester improved osteogenic properties of PBS (PBSGL0) fibers in a PGL concentration-related manner. The increase in osteoblastic gene expression and relevant proteins, as well as ALP activity and calcium content, with increasing PGL ratio indicates that the presence of the glycolate group improves the osteogenic differentiation of rabbit MSCs in a concentration-dependent manner on PBSGL_n_ electrospun fibers (Fig. 9). *In vivo* results of the current study also confirmed that more bone formation was observed on PBSGL_n_ membranes with a higher glycolate content (Fig. 10).

While collagen-based, resorbable membrane materials, such as Bio-Gide, are well-accepted by clinicians for bone regeneration because of their high biocompatibility and the fact that they do not require a second surgery, they have exhibited poor mechanical strength and tend to collapse before the bone has sufficiently healed^17,20,21^. It is worth mentioning that because of the heterogeneity existed between collagenous membranes, as we discussed earlier, Bio-Gide membrane is not necessarily representative of all collagenous membranes. An alternative to these resorbable materials are PBSGL_n_ membranes whose physical and mechanical properties are tunable to the natural bone regeneration process and can exclude the faster-growing connective tissues from a wound region for a designated period. This would allow slower-growing bone tissues to occupy the defect. Although the gold standard Bio-Gide membrane still exhibits higher bone formation than PBSGL_n_, these new membranes show great promise for future applications because of their tunable degradation rate, better mechanical properties, and superior space maintenance. Future studies are needed to evaluate PBSGL_n_’s ability to prevent microbial contamination during transmucosal/transgingival healing and promote periodontal regeneration.

The purpose of this study was to synthesize a novel PBSGL_n_ co-polyester with favorable physicomechanical properties and to investigate the *in vitro* and *in vivo* biocompatibility and osteoconductivity of the electrospun PBSGL_n_ nanofibers as GBR membranes for periodontal applications. The resultant co-polyesters possessed high intrinsic viscosity, high molecular weight, and low levels of carboxylic groups, demonstrating an appropriate polymerization route. Biocompatibility results showed an increase in rabbit MSC metabolic activity with increasing glycolate ratio of the electrospun PBSGL_n_ nanofibers. Based on real-time quantitative PCR results, osteogenic-related genes expressed in seeded PBSGL_n_ membranes increased with increasing glycolate concentration. Results of calcium content and Alizarin Red staining also confirmed that mineral deposition was higher for those groups with higher amounts of glycolate.

Subsequently, four electrospun PBSGL_n_ membranes were implanted into rabbit calvarial defects to evaluate their osteoconductivities. It was found that PBSGL_n_ membranes with a higher ratio of PGL were more osteoconductive. Overall, this study introduced a novel co-polyester with tunable mechanical properties suitable for GBR in the periodontal application which deals with the reconstruction of bone alone in an edentulous intraoral site. Guided tissue regeneration (GTR) application of this co-polyester—which encompasses the reconstruction of all the missing tissues of the periodontium that are destroyed secondary to periodontitis (*i.e*. the regeneration of new bone, cementum, and periodontal ligament on a previously diseased root surface)—needs to be evaluated in future studies. In addition, to translate this model from a rabbit calvarial critical defect model to human application, more future studies need to be performed to evaluate the effects of oral bacterial exposure on the function of the PBSGL membranes and potential surgical morbidities in large animal models.

## Methods

### Materials

Butylene glycol (BG), succinic acid (SA), titanium butoxide (TBT) as the poly-condensation catalyst, phosphoric acid (PA), and chloroform, were purchased from Merck Co. (Darmstadt, Germany). Glycolide (GL) and hexafluoroisopropanol, as an electrospinning solvent, were purchased from Alfa Aesar (Massachusetts, USA). Dulbecco’s modified Eagle’s medium (DMEM), fetal bovine serum (FBS), Dulbecco’s phosphate buffer saline (DPBS), streptomycin, penicillin G, amphotericin B, trypsin, and ethylenediaminetetraacetic acid (EDTA) were bought from Gibco (Life Technologies, Carlsbad, CA, USA). Quant-it PicoGreen dsDNA Assay Kit was bought from Invitrogen (Carlsbad, CA, USA). Alizarin Red S, dimethylsulfoxide (DMSO), paraformaldehyde, tin 2-ethylhexanoate (Sn(Oct)_2_), glycerolphosphate, ascorbic acid, dexamethasone, RIPA buffer, protease inhibitor cocktail, QuantiPro BCA Assay Kit, CDCl_3_, tetramethylsilane (TMS), and 4′,6-diamidino-2-phenylindole (DAPI) were obtained from Sigma-Aldrich (St. Louis, MO, USA). The QuantiChrom Alkaline Phosphatase Assay Kit and QuantiChrom Calcium Assay Kit were purchased from Bioassay Systems (Hayward, CA, USA). The CellTiter 96 AQ_ueous_ One Solution Cell Proliferation Assay (MTS) was bought from Promega (Madison, WI, USA). Hybrid-R RNA extraction kit was purchased from GeneAll (Seoul, Korea). DNase I and the Haematoxylin and Eosin Stain Kit were obtained from Thermo Fisher Scientific (Hamburg, Germany). PrimeScript RT reagent Kit was received from TaKaRa (Shiga, Japan). SYBR Green PCR Master Mix was bought from Applied Biosystems (Foster City, CA, USA). Mouse anti-rabbit vinculin, osteocalcin (OCN), collagen type 1 and β actin primary antibodies as well as goat anti-mouse secondary antibody were obtained from Abcam (Cambridge, MA, USA). Western Blotting Luminol Reagent and Blotto solution were purchased from Santa Cruz Biotechnology (Santa Cruz, CA, USA). 7.5% Mini-PROTEAN TGX Precast Protein Gels were received from Bio-Rad Laboratories, Inc. (Hercules, CA, USA). All the forward and reverse primers were obtained from Integrated DNA Technologies, Inc. (Coralville, IA, USA). All solvents were obtained at lab-grade purity and used without further purification or processing.

### Synthesis of co-polyesters

**Figure 11:**
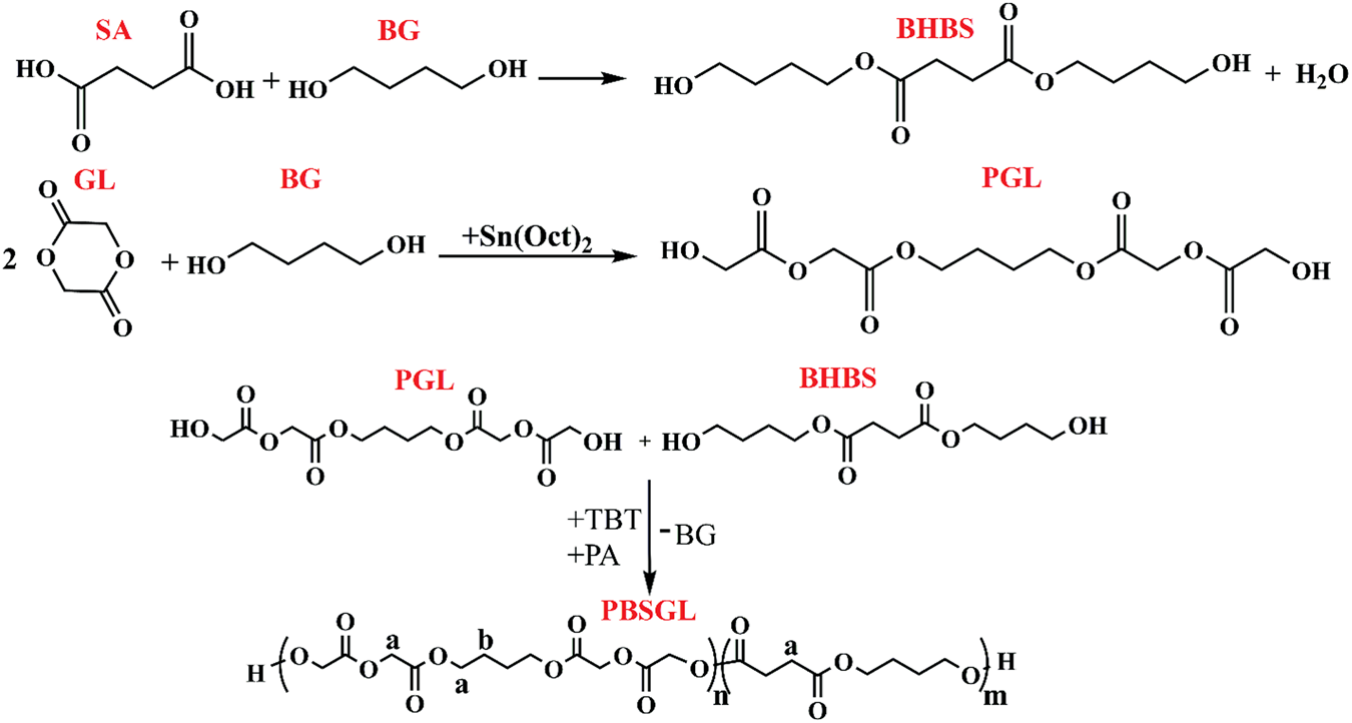
Reactions during two-step synthesis of PBSGL co-polyesters: direct esterification of SA and BG for synthesis of BHBS; ring opening polymerization of GL and BG in the presence of Sn(Oct)_2_ as a catalyst to produce short chain PGL; condensation reaction between PGL and BHBS in the presence of TBT (catalyst) and PA (thermal stabilizer) for the synthesis of PBSGL co-polyesters.

All co-polymers were synthesized using two-step polymerization. First, GL and SA were reacted with BG separately to produce Bis(4-hydroxybutyl) succinate (BHBS) and short chain polyglycolic acid (PGL), respectively. BHBS was synthesized as described previously^63^. GL and BG were mixed with a 2:1 molar ratio of GL to BG; this solution was subsequently poured into the reactor as we previously described^63^ to synthesize PGL. The paste was dried for 30 min at 110 °C under a N_2_ atmosphere. Then, Sn(Oct)_2_ was added as catalyst and the temperature was increased to 150 °C to start polymerization. This step was continued for about 12 h. Finally, BHBS and PGL monomers with predetermined mole ratios, 200 ppm TBT (as catalyst), and 12 ppm phosphoric acid (as thermal stabilizer), were mixed in the reactor for 10 min at 170 °C and a N_2_ atmosphere to prepare poly (butylene succinate-co-glycolic acid) (PBSGL). Under vacuum, the temperature was then increased to 238 °C to start the poly-condensation step, which continued for about 3 h until the mixer’s torque reached the appropriate value. Figure 11 demonstrates the reactions of the synthesis mechanism of the co-polyesters.

### Fiber matrix fabrication

The electrospinning solutions were prepared by dissolving the polymers in hexafluoroisopropanol. The pre-described electrospinning setup was used to fabricate non-woven fiber matrices at room temperature and pressure^64^. Electrospinning solution concentration (C) in the range of 8–12 wt%, applied voltage (V) in the range of 16–22 kV, injection rate (Q) of 400 µL/min, and needle-to-collector distance (D) in the range of 6–9 cm were adjusted to minimize the fiber diameter. Electrospun fibers were vacuum dried overnight at ambient temperature.

### Characterization

The intrinsic viscosity of the samples dissolved in chloroform was measured using a Ubbelohde capillary viscometer at 25 ± 0.1 °C (0c type, Lauda, Lauda-Königshofen, Germany). The following equation was used to approximate the number average molecular weight (M_n_) of the samples^65^:

6 $${\bar{M}}_{n}=3.29\times {[\eta ]}^{1.54}$$

^1^H-NMR spectra were recorded on a Varian Mercury-300 HNMR (Varian, Palo Alto, CA, USA) at ambient temperature. CDCl_3_ and TMS were used as the solvent and reference, respectively.

A Q-2000 DSC was used to measure thermal properties of the samples (TA instruments, New Castle, DE, USA). First, 5–8 mg of the samples were heated to 160 °C to remove the thermal history. Then, the samples were cooled to −80 °C and heated to 160 °C at the rate of 10 °C/min.

An Equinox wide-angle XRD (Model 3000, Bruker, Madison, WI, USA) equipped with a CuKα1 radiation source (λ = 0.1541874 nm) was used to measure the diffraction pattern with 2θ angles ranging from 4 to 120° and to further investigate the crystallinity of the samples. The samples were then hot pressed, using a Carver press (Wabash, IN, USA) at 120 °C to produce thin sheets (20 × 20 × 0.1 mm), which were then cooled to ambient temperature at a rate of 5 °C/min.

A VEGA3 SBU variable pressure SEM (Tescan, Kotoutovice, Czech Republic) was used to obtain images of the nanofiber samples at an accelerating voltage of 8 keV. A Denton Desk II sputter coater (Moorestown, NJ, USA) was used to coat samples with gold.

Tensile properties of the electrospun fiber matrices were measured at room temperature with an Instron material testing machine (Model 5543 A, Norwood, MA, USA), with a crosshead speed of 5 mm/min. The wettability (contact angle) of each PBSGL_n_ nanofibrous membrane was evaluated by applying the sessile drop technique (with distilled water) using a VCA Optima Surface Analysis system (AST products, Inc., Billerica, MA, USA) at ambient conditions. *In vitro* mass loss of the PBSGL_n_ membranes was measured after 60 days, as we previously described^66^.

### Cell culture

Bone marrow-derived MSCs from the femur of a 3.5 kg, male New Zealand white rabbit at skeletal maturity were ordered from the National Cell Bank, Pasteur Institute of Iran. The basal culture medium was DMEM supplemented with 10% FBS, 100 U/ml penicillin G, 100 µg/ml streptomycin, and 0.25 mg/ml amphotericin B (pH 7.2).

### Metabolic activity (proliferation)

The rabbit bone marrow-derived MSC metabolic activity (proliferation) on each PBSGL membrane (3 cm × 3 cm) was evaluated using a colorimetric 3-(4, 5-dimethylthiazol-2-yl)-5-(3-carboxymethoxyphenyl)-2(4-sulfophenyl)-2H tetrazolium (MTS) assay (Promega, Madison, WI, USA), as we previously described^66^. Briefly, PBSGL membranes were sterilized by soaking in 100× sterilizing solution containing penicillin G (10,000 U/ml), streptomycin (10,000 µg/ml), and amphotericin B (250 µg/ml) overnight. PBSGL membranes were then washed 3× with PBS solution and placed in basal medium for 1 h prior to the cell seeding. MSCs were seeded on the electrospun nanofibers at a density of 4,000 cells per cm^2^. At each time point (1, 3, 7 and 12 days), membranes were transferred to new plates in order to remove the effect of cells grown on the plates and incubated in 20% MTS solution in serum-free medium at 37 °C for 3 h. The absorbance at 490 nm of the supernatant media was measured using a spectrophotometric plate reader (FLUOstar Omega, BMG Lab Technologies, Offenburg, Germany), and the absorbance was correlated to the number of rabbit MSCs^66^.

### Cell differentiation

Rabbit MSCs (4000 cells per cm^2^) were seeded on each sterile membrane and allowed to adhere to the surface of the nanofibers by incubating for 8 h at 37 °C and 5% CO_2_ in basal medium. Then, the basal medium was removed and replaced with osteogenic medium (basal medium supplemented with 10 mM β-glycerolphosphate, 0.2 mM ascorbic acid, and 10^−8^ M dexamethasone). The osteogenic medium was refreshed every 3 days for 21 days. Rabbit MSCs (with the same cell density, which was adjusted in accordance with the specific surface area of the materials) cultured in osteogenic medium in cell culture 6-well plates were used as control groups.

### RNA extraction and cDNA synthesis

At each time point (1, 7, 14, and 21 days), the total RNA was extracted from MSC-seeded membranes using a Hybrid-R RNA extraction kit (GeneAll, Seoul, Korea). The extraction was performed according to the kit’s manual. DNase I from Thermo Fisher Scientific (Hamburg, Germany) was used to eliminate genomic DNA following the manufacturer’s manual. The obtained RNA for different groups was then normalized based on their RNA content, obtained by a NanoDrop spectrophotometer (ND-1000, Thermo Fisher Scientific, MA, USA). The cDNA synthesis was performed using a PrimeScript RT reagent Kit (TaKaRa, Shiga, Japan) following the kit’s procedure, using a Genius thermocycler (Techne, Cambridge, UK).

### Gene expression

Gene expression was assessed using a real-time polymerase chain reaction (qRT-PCT) kit with SYBR Green PCR Master Mix (Applied Biosystems, Foster City, CA, USA), as we previously described^4,66^.

Primers included GAPDH (housekeeping; 5′-CGTCTGCCCTATCAACTTTCG-3′, 5′-GTTTCTCAGGCTCCCTCT-3′), OCN (late marker of osteogenesis; 5′-GACACCATGAGGACCCTCTC-3′, 5′-GCCTGGTAGTTGTTGTGAGC-3′), RUNX2 (early marker of osteogenic differentiation; 5′-GGAGTGGACGAGGCAAGAGT-3′, 5′-AGGCGGTCAGAGAACAAACTAGG-3), and Col1α1 (early pre-osteogenic marker; 5′-GCGGTGGTTACGACTTTGGTT-3′, 5′-AGTGAGGAGGGTCTCAATCTG-3). Fold change expression of each target gene was calculated by applying the 2^−∆∆CT^method.

### Biochemical assays

At each time point (1, 7, 14 and 21 days), the osteogenic medium was drained, and in order to remove serum proteins, MSC-seeded PBSGL membranes were submerged in serum-free DMEM for 8 h, followed by triple DPBS washing. The rabbit MSCs were then lysed with lysis buffer (10 mM Tris supplemented with 0.2% triton in PBS). This lysate was used for measuring the content of DNA and calcium as well as ALP activity using the PicoGreen DNA assay, and QuantiChrom Calcium and ALP assay kits, respectively, according to the manufacturer’s instructions. ALP activity and calcium contents were normalized based on the DNA contents of each sample at each time point.

### Western blotting

Protein expression of markers specific to bone cells, including OCN and collagen type 1-α1 at day 21 was quantified *via* Western blotting, as we previously described^66^. Briefly, after removal of serum protein as described in biochemical assays section, rabbit MSC-seeded membranes were treated with a RIPA buffer supplemented with a protease inhibitor cocktail to lyse the cells. Lysates with the same amount of total protein (measured by a BCA kit) were run through 7.5% SDS-PAGE gel. Then, their separated proteins were transferred to a nitrocellulose membrane. After blocking in Blotto solution, nitrocellulose membranes were incubated in primary and horseradish peroxidase (HRP) conjugated secondary antibodies. Western blotting luminol reagent was used to visualize the target proteins; their bands were imaged using a Bio-Rad ChemiDoc MP System, and quantified by ImageJ software (NIH, Bethesda, MD, USA).

### Animal study

Two time points (one and two months after surgery), each one using five adult male white New Zealand rabbits weighing approximately 3 Kg, were selected for *in vivo* evaluation of new bone formation for different groups. All the experimental protocols were performed according to the approved procedures for experimental animals by the Ethical Committee in Animal Research from the Veterinary Medicine College at University of Tehran, which were extensively explained in our previous work^4^. Briefly, four circular defects with the same size (6 mm diameter) were made in the calvaria of rabbits by removing the external cortical plate after elevation of full-thickness flap including periosteum. After covering the defects randomly by PBSGL membranes following suturing the periosteum and skin, post-surgical monitoring and care were performed for all animals, which were kept in separate cages. At each time point, five animals were sacrificed using thiopental sodium (Nesdonal, Specia, Paris, France) saturated solution, and six serial sections of equal thickness with the highest diameter from the center of each defect were stained with haematoxylin and eosin and were evaluated under an Olympus light microscope (Olympus SZX9, Olympus Optical, Kawasaki, Japan) by a pathologist to grade the immune response. Images with the largest defect diameter were captured under 100× magnification by a digital camera (Nikon Eclipse E8400, Nikon, Kawasaki, Japan), and were used to evaluate the amount of new bone formation using a histometry software program^4^.

### Statistical analysis

Power analysis was used to determine the sample size for *in vivo* study, according to our previous work^4^. For statistical evaluation of the differences in the amount of new bone formation (by percent) between the groups, based on a power of 90% and a significance level of 0.05 (two-tailed), a sample size of five was determined to be adequate.

For determining the interaction effect between time points and the surgical groups, as well as to compare the amount of new bone formation in four surgical groups, a two-way repeated measure ANOVA was performed. The nonparametric Friedman test was conducted to compare the extent of inflammation in the surgical areas at the two time points. All values presented herein are expressed as mean ± standard deviation. Each experiment was performed in triplicate. To determine statistical differences for *in vitro* experiments, a two-way ANOVA analysis with replication test was used, followed by a Student’s two-tailed t-test. P values less than 0.05 were considered statistically significant.
